# An unexpected finding of cardiac injury penetrating the diaphragm in a suspected abdominal trauma case

**DOI:** 10.1093/jscr/rjaf586

**Published:** 2025-08-06

**Authors:** Shingo Kunioka, Fumiaki Kimura, Hideki Isa, Kentaro Shirakura, Shutaro Wachi, Daiki Sunada, Naohiro Kokita, Motoi Okada, Katsuaki Magishi, Yuichi Izumi, Hiroyuki Kamiya

**Affiliations:** Department of Cardiovascular Surgery, Nayoro City General Hospital, Nishi-7-Minami-8-1, Nayoro, Hokkaido 096-8511, Japan; Department of Cardiac Surgery, Asahikawa Medical University, Midorigaoka Higashi 2-1-1-1, Asahikawa 078-8510, Japan; Department of Emergency Medicine, Asahikawa Medical University, Midorigaoka Higashi 2-1-1-1, Asahikawa 078-8510, Japan; Department of Cardiovascular Surgery, Nayoro City General Hospital, Nishi-7-Minami-8-1, Nayoro, Hokkaido 096-8511, Japan; Department of Cardiovascular Surgery, Nayoro City General Hospital, Nishi-7-Minami-8-1, Nayoro, Hokkaido 096-8511, Japan; Department of Cardiovascular Surgery, Nayoro City General Hospital, Nishi-7-Minami-8-1, Nayoro, Hokkaido 096-8511, Japan; Department of Emergency and Critical Care Center, Nayoro City General Hospital, Nishi-7-Minami-8-1, Nayoro, Hokkaido 096-8511, Japan; Department of Emergency and Critical Care Center, Nayoro City General Hospital, Nishi-7-Minami-8-1, Nayoro, Hokkaido 096-8511, Japan; Department of Emergency Medicine, Asahikawa Medical University, Midorigaoka Higashi 2-1-1-1, Asahikawa 078-8510, Japan; Department of Emergency Medicine, Asahikawa Medical University, Midorigaoka Higashi 2-1-1-1, Asahikawa 078-8510, Japan; Department of Cardiovascular Surgery, Nayoro City General Hospital, Nishi-7-Minami-8-1, Nayoro, Hokkaido 096-8511, Japan; Department of Cardiovascular Surgery, Nayoro City General Hospital, Nishi-7-Minami-8-1, Nayoro, Hokkaido 096-8511, Japan; Department of Cardiac Surgery, Asahikawa Medical University, Midorigaoka Higashi 2-1-1-1, Asahikawa 078-8510, Japan

**Keywords:** penetrating cardiac injury, junctional zone trauma, diaphragmatic injury, right ventricular stab wound, elderly trauma

## Abstract

We report a rare case of penetrating abdominal trauma involving right ventricular injury. An 81-year-old man presented with a stab wound in the upper abdomen and hemodynamic instability. Hemorrhagic shock was initially suspected to be due to intra-abdominal organ damage. Emergency laparotomy revealed no evidence of intra-abdominal organ injury; however, subsequent thoracotomy and sternotomy identified cardiac tamponade from right ventricular injury caused by a penetrating object that traversed the diaphragm. Prompt surgical intervention enabled successful resuscitation. This case highlights the diagnostic challenge of “junctional zone” trauma, where both thoracic and abdominal organs may be involved. Despite advanced age, the patient was discharged in good condition.

## Introduction

Penetrating cardiac injury, most commonly resulting from gunshot or stab wounds, represents one of the most lethal forms of trauma, with prehospital mortality rates exceeding 80% [1]. The risk is particularly elevated in elderly individuals due to diminished physiological reserves and increased vulnerability to delayed intervention [2]. The anterior chest is the most frequently reported site of entry for penetrating injuries involving the heart and aorta. Sauer and Murdock [3] described the “danger zone” as an anatomical region where penetrating injuries should prompt strong suspicion of cardiac or aortic trauma. In contrast, penetrating injuries occurring below the nipple line are more often associated with potential intra-abdominal organ damage. However, the anatomical region between the nipple line and the umbilicus—commonly referred to as the “junctional zone”—is especially diagnostically challenging, as injuries in this area may traverse the diaphragm and lead to missed intrathoracic injuries, including cardiac trauma [4].

Here, we report a rare case of penetrating abdominal trauma in which hemorrhagic shock was initially attributed to suspected intra-abdominal organ injury. However, emergency surgical exploration revealed that the penetrating object had traversed the diaphragm and resulted in right ventricular injury. The patient, who presented with hemodynamic instability, was successfully resuscitated through timely surgical intervention.

## Case report

**Figure 1:**
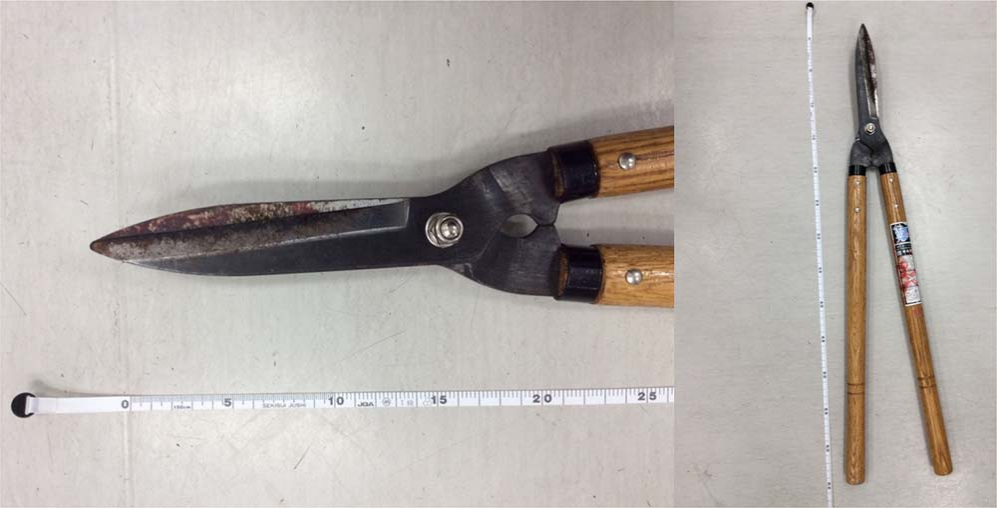
The pruning shears that caused the stab wound in this case. The pruning shears responsible for the injury, with a blade length of ~15 cm. Blood clots were visible up to the base of the blade, indicating full-length penetration.

An 81-year-old man was found collapsed in his garden, prompting an emergency call by a bystander. A 40-mm incised wound was observed in the left upper abdomen, and it was suspected that he had accidentally stabbed himself with pruning shears while working in the garden (Fig. 1). By the time emergency personnel arrived, the shears had already been removed, and the incident had not been witnessed. Thus, the angle and depth of penetration remained unclear. Given the location of the wound, hemorrhagic shock due to intra-abdominal organ injury was initially suspected. The patient was transported to our hospital by ambulance, intubated upon arrival, and brought directly to the operating room, as he exhibited a thready pulse and was judged to be in impending cardiac arrest due to hemorrhagic shock.

**Figure 2:**
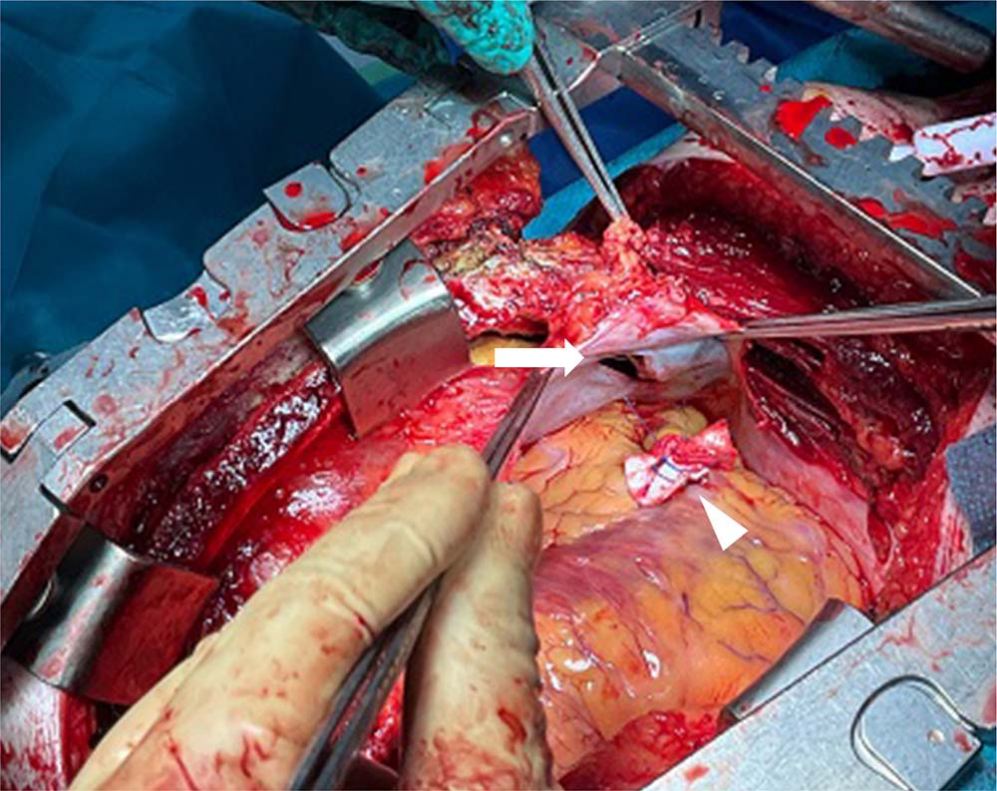
Intraoperative findings. The pruning implement had penetrated the diaphragm (arrow), resulting in right ventricular injury. Hemostasis was achieved with 3-0 polypropylene sutures reinforced with pledgets (arrowhead).

**Figure 3:**
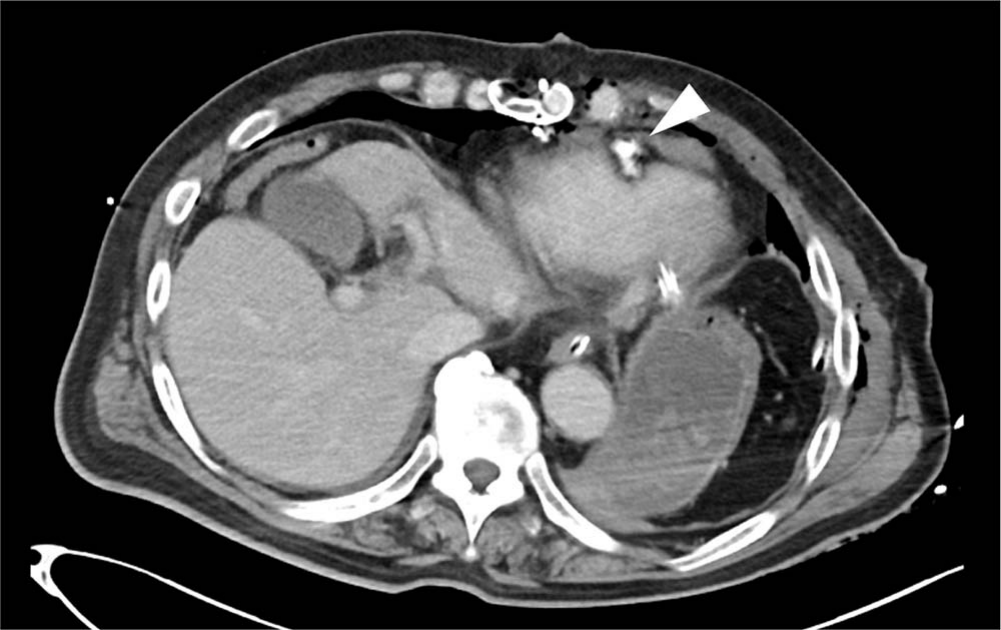
Postoperative CT findings. No evidence of pericardial effusion was observed, and the surgical site was hemostatic (arrowhead).

The external appearance of the wound did not clearly reflected the extent or nature of internal injury. Based on the presumptive diagnosis of intra-abdominal injury, an emergency exploratory laparotomy was performed. The initial exploration by general surgeons revealed no evidence of intra-abdominal organ damage. Given the suspicion of diaphragmatic penetration and intrathoracic hemorrhage, the cardiac surgery team proceeded with a left thoracotomy. This revealed dark, reddish fluid suggestive of pericardial hemorrhage. Suspecting obstructive shock due to cardiac tamponade, the surgical approach was converted to a median sternotomy. Upon opening the pericardium, a significant hemopericardium was identified, and active bleeding from the anterior wall of the right ventricle was successfully controlled using 3-0 polypropylene sutures with pledgets (Fig. 2). Standard chest closure and wound closure were subsequently performed, and the patient was transferred to the intensive care unit in a stable condition. The postoperative course was uneventful. Follow-up computed tomography (CT) imaging demonstrated no pericardial effusion or other complications (Fig. 3). The patient recovered favorably and was discharged home one month after surgery.

## Discussion

This case report holds significance in two key aspects. First, despite the patient’s advanced age—being over 80 years old—and the prolonged transport time due to the rural location, the availability of a physician-staffed emergency vehicle enabled prompt surgical intervention and successful resuscitation. Second, the case highlights the diagnostic complexity of penetrating trauma, particularly when the injury occurs in the “junctional zone,” where both thoracic and abdominal involvement must be carefully considered.

Penetrating cardiac injuries carry a prehospital mortality rate of 80%–90%, and timely surgical intervention is essential for survival [1]. Baxi *et al.* [5] reported that, among patients with penetrating cardiac injuries, the presence of cardiac tamponade, right ventricular injury, and injury confined to a single cardiac chamber are associated with improved survival. Conversely, Lee *et al.* [2] demonstrated that trauma patients aged 65 years or older have a 4.6-fold higher risk of in-hospital mortality compared to younger individuals. In the present case, the patient sustained a right ventricular injury complicated by cardiac tamponade, yet he reached the operating room without experiencing cardiac arrest—a factor generally associated with a favorable prognosis. Notably, the patient survived to discharge despite being over 80 years old, underscoring the exceptional nature of this case. Given the high lethality of such injuries, rapid transport and immediate surgical intervention are strongly recommended. In our case, emergency surgery was initiated immediately upon arrival, facilitating successful resuscitation [1].

**Figure 4:**
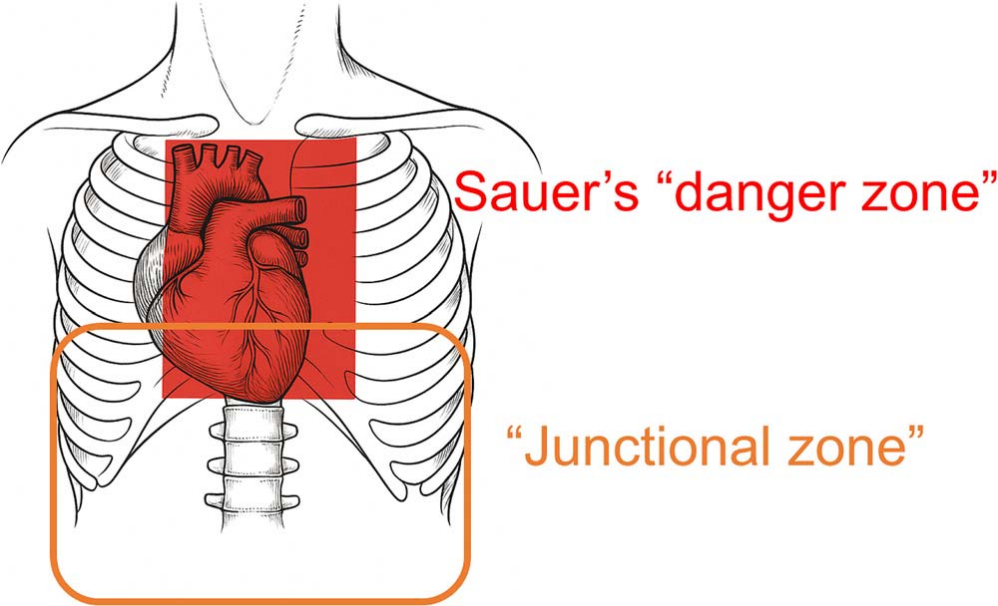
Assessment of organ injuries based on the stab wound entry site. In cases where the stab wound is located within Sauer’s danger zone, injuries to the heart or aorta should be suspected. If the entry point is in the junctional zone, transdiaphragmatic injuries must also be considered.

Furthermore, this case is particularly notable because the suspected site of injury, based on the abdominal entry wound, differed from the actual site of life-threatening trauma. In cases such as this—where the patient is hemodynamically unstable and immediate surgical exploration is required—there is a risk that the exact cause of hemorrhagic shock may not be initially identified. Although intra-abdominal organ injury was suspected based on the entry site, the true diagnosis was a penetrating cardiac injury that had traversed the diaphragm. This case underscores the diagnostic challenges inherent in managing junctional zone trauma. In addition to the well-recognized “danger zone” described by Sauer for thoracic penetrating injuries [3], clinicians must remain vigilant for the possibility of thoracic organ injury—including diaphragmatic and cardiac involvement—even when the entry wound is located in the upper abdomen. This region, referred to as the “junctional zone,” is known to pose a high risk of missed diagnoses. Likewise, in cases of thoracic penetrating trauma, clinicians must also consider the potential for intra-abdominal organ injury via diaphragmatic penetration (Fig. 4).

In conclusion, this is a rare case of penetrating trauma with an abdominal entry site that traversed the diaphragm and resulted in right ventricular injury. Despite the patient’s advanced age, prompt surgical management led to survival. This case emphasizes the diagnostic challenges associated with abdominal stab wounds and highlights the importance of considering junctional zone injury patterns in trauma assessment.

## Data Availability

Although data evaluated in the present report is not available in a public repository, data will be made available to other researchers upon reasonable request.
